# Trauma research in the Baltic countries: from political oppression to recovery

**DOI:** 10.3402/ejpt.v7.29295

**Published:** 2016-03-18

**Authors:** Evaldas Kazlauskas, Paulina Zelviene

**Affiliations:** Department of Clinical and Organizational Psychology, Vilnius University, Vilnius, Lithuania

**Keywords:** Posttraumatic stress, review, trauma, Baltic, Lithuania, Latvia, Estonia

## Abstract

The aim of this paper is to provide an overview of traumatic stress studies from the three Baltic countries—Lithuania, Latvia, and Estonia—and reveal how specific social context contributes to the topics relevant in traumatic stress field in the region. Traumatic stress studies in the Baltic countries are closely related to the complicated history of the region. It was only since the restoration of independence of the Baltic States in the 1990s when traumatic stress studies could emerge. The start of the psychotraumatology in the Baltic States was inspired by the interest of the psychological effects of political violence. Four major topics in traumatic stress literature from the Baltic countries were identified in this article: political violence studies, epidemiology of trauma and posttraumatic stress disorder (PTSD), disaster studies, and developmental aspects of trauma. Traumatic events prevalence was reported between 70 and 75%, and PTSD prevalence range 2–7% in the Baltic countries. The interest in psychotraumatology in the Baltic countries is rising.

Lithuania, Latvia, and Estonia—the three Baltic countries (also known as *the Baltic States*, or *the Baltics*)—are located on the opposite coast of the Baltic Sea from Sweden and Finland, on the Northeast edge of the European Union (EU), between Russia and other EU countries. With a population of about 6 million, the region has undergone significant social changes during the last few decades. The joint history of the Baltic countries started in 1918 after World War I when the new countries, including Lithuania, Latvia, and Estonia, appeared on the map of Europe. Development of the independent Baltic countries was ruined by World War II (WW2)-related events two decades later. At the beginning of the WW2 in 1940, the Soviet Army entered the Baltic countries, resulting in the annexation of the Baltics and proclamation of the three new Soviet Republics as a part of the Soviet Union. Nazi Germany attacked the Soviet Union in 1941 and the occupation of the Baltic countries by the Nazis lasted from 1941 to 1944. In 1944, the Soviet Army re-entered the Baltic States moving the Eastern front towards Germany. After WW2, the Baltic countries remained occupied and incorporated into the Soviet Union for almost 50 years. During the Soviet period, the Baltic countries were marked with prolonged political oppression, with hundreds of thousands of people exposed to political violence, political imprisonment, and forced displacement. In the 1990s, the Baltic countries declared independence, which was followed by the joining of the EU and NATO in 2004.

## The rise of interest in traumatic stress studies in the Baltic States

The social and political context shaped the theories and research of traumatic stress in Europe (Weisaeth, 2002), with the Baltic countries being no exception in that. The history of the Baltic States is closely related to the development of the field of traumatic stress in these countries. Psychology and psychotherapy as a profession and research field was oppressed for decades during the Soviet regime (Bagdonas, Pociūtė, Rimkutė, & Valickas, 2008; Draguns, 2006; Reņg‘e & Draguns, 2012). In 1980, when the diagnosis of posttraumatic stress disorder (PTSD) was included in the *Diagnostic and Statistical Manual of Mental Disorders III* (DSM-III), the Soviet regime in the Baltic countries still ruled, and no acknowledgment of trauma and stress-related disorders was possible. To make things worse, it is also well documented that psychiatry in the Soviet Union was widely used as part of an oppressive system to persecute those individuals in opposition to the Soviet regime (Van Voren, 2010).

It was only after the restoration of the independence of the Baltic countries in the 1990s and the collapse of the Soviet Union that trauma research and practice could emerge (Bagdonas et al., 2008; Draguns, 2006). A significant development in the field of traumatic stress in the Baltic States was the first international conference on the effects of political violence in 2003 hosted by Vilnius University, and a publication of the first English language book edited by Gailienė “The Psychology of Extreme Traumatisation” in 2005. The rise in the interest of psychotraumatology in the region was further facilitated by a successful and very well attended Annual Vilnius Trauma Psychology conferences starting from 2010, with keynote contributions from leading experts from Europe, including B. P. R. Gersons, M. H. van IJzendoorn, A. Maercker, W. Yule, and others. These conferences inspired the foundation of the Lithuanian Society for Traumatic Stress Studies (LSTSS) in 2013. LSTSS in cooperation with the European Society for Traumatic Stress studies organized the 14th ESTSS Conference hosted by Vilnius University, Lithuania, in 2015, which was a significant input on the further development of psychotraumatology in the Baltic countries.

## Traumatic stress research in the Baltic countries

The aim of this article is to provide a comprehensive overview on traumatic stress studies from the three Baltic countries and reveal how specific social context contributes to the topics relevant in traumatic stress field in the region based on the available English language publications from the region. We could identify four major topics in Baltic psychotraumatology: political violence studies, epidemiology of trauma and PTSD, disaster studies, and developmental studies.

### Political violence studies

Psychotraumatology in the Baltic States was grounded on the interest of the psychological effects of political violence and oppression. One of the very first PTSD studies was published in 1992 soon after restoration of independence of Lithuania by Griškonyté, Barzdžiukaité, and Ručinskiené (1992) on posttraumatic reactions in a clinical sample of 27 survivors of the Soviet army's military violence against a peaceful demonstration on January 13, 1991, in Vilnius, Lithuania. The long-term psychological effects of 1991 “Bloody January” on survivors and relatives of the deceased were explored 20 years later in a qualitative study by Povilaitis et al. (2015). This study, which was focused on coping and the perceived impact on survivors, found that social support and other psychosocial factors, including memorials, social recognition, and debates about social status of survivors, are significant for coping with a traumatic injury or loss related to political violence of January 1991 (Povilaitis et al., 2015).

Two large-scale studies on the long-term effects of political oppression were conducted in Latvia and Lithuania. Vidnere and Nucho (2000) contacted about 600 Latvian ex-deportees in a self-report survey that focused on the traumatic experiences and coping of Latvian survivors of forced displacement. The study revealed a complicated long-term traumatization of survivors, which also included persecutions and restrictions after their return to Latvia. Latvian survivors indicated the importance of support from other survivors (39%) and religion (50%) in coping with political violence. Similar results to the Latvian study were also found in a Lithuanian study on the long-term effects of political violence (Gailienė & Kazlauskas, 2005; Kazlauskas, 2006). Results from about 1,400 Lithuanian survivors of political violence indicated long-term health and mental health issues related with the exposure to political violence, almost 50% reported flashbacks and 33% reported nightmares at the time of the study (Gailienė & Kazlauskas, 2005). Mutual support between survivors, hope, and religion was very important for surviving the harsh conditions of displacement and political imprisonment, and political activity was reported as an important resource of resilience (Kazlauskas, 2006).

Research on the long-term effects of political violence on survivors in Lithuania was followed by intergenerational studies. In her doctoral dissertation, Vaskelienė (2012) found no significant clinical effects of parental exposure to Soviet political violence in the offspring in comparison with the control group and described family secrecy as an important aspect of communication in families who were exposed to political oppression. Kazlauskas and Želvienė (2015) reported interesting results exploring the resiliency of family members coming from families with a history of political violence. The study found better psychological well-being and higher resilience in families with a history of political violence in Lithuania in comparison with a group from families without a history of political oppression (Kazlauskas & Želvienė, 2015).

Domanskaitė-Gota, Gailienė, and Girdziušaitė (2005) analyzed Afghanistan war (1979–1989) veterans’ traumatic stress reactions and risk factors in the context of Soviet political oppression. Domanskaitė-Gota et al. (2005) found a 13.5% PTSD prevalence in the sample of 174 Afghanistan war veterans based on DSM-IV criteria. Domanskaitė-Gota (2014) described how the oppressive regime recruited young men to fight in the Afghanistan war, with secrecy around the process of recruitment and forced signing of war-related information non-disclosure agreements. Further analysis in the doctoral dissertation of Domanskaitė-Gota (2014) revealed that PTSD reactions in the Lithuanian Afghanistan war veterans group were predicted by adaptation problems after deployment, lack of psychosocial support from friends and family, and higher rates of alcohol consumption.

### Prevalence of trauma and PTSD

A review of epidemiological data on mental health disorders in Europe reveals a 12-month prevalence of PTSD ranging from 1.1 to 2.9%, with a significant decline in PTSD prevalence with age (Wittchen et al., 2011). Several studies provided epidemiological data in Lithuania with a reported tendency of higher trauma and PTSD prevalence rates in comparison to Europe in general. Kazlauskas and Želvienė (2015) reported a 70% prevalence of exposure to at least one life-time traumatic event in the Lithuanian general population based on a sample of 626 adults aged from 18 to 89. Domanskaité-Gota, Elklit, and Christiansen (2009) reported a 75% prevalence of traumatic experiences in the 15-year-old representative sample of 183 adolescents from Lithuania, and a 7% prevalence of PTSD based on DSM-IV criteria. The prevalence of PTSD was 2% in the sample of 998 primary care patients in Lithuania (Bunevicius et al., 2014) using the Mini International Neuropsychiatric Interview (MINI) based on ICD-10 criteria. Epidemiological data on the prevalence of PTSD in Latvia and Estonia are lacking.

### Disaster studies

At least two major disasters struck the region: the Chernobyl Nuclear Power Station accident in 1986 and MS Estonia shipwreck disaster in 1994.

The Chernobyl accident is considered to be the biggest nuclear disaster globally. There are some studies of the health effects, mortality, and suicide ideations in a sample of Chernobyl disaster cleanup workers from Estonia (Rahu, Rahu, Tekkel, & Bromet, 2006; Rahu et al., 1997), but little is known about the posttraumatic effects of the disaster in the Baltic countries. Povilaitienė, Auškalnytė, Grigienė, and Skruibis (2015) only recently published a qualitative study which reveals the complicated adjustment of Lithuanian Chernobyl cleanup workers. The most difficult experience was powerlessness and uncertainties related with unknown doses of radiation exposure, and consequences of radiation on health. The lack of social acknowledgment was predominant in the interviews with survivors (Povilaitienė et al., 2015).

MS *Estonia* ferry sank rapidly when she was crossing the Baltic Sea from Tallinn to Stockholm resulting in 852 deaths, and only 137 were saved. The first psychological effects of the *Estonia* accident were reported in 1996, and prospective study results were published 14 and 15 years after the disaster in a Swedish sample of survivors (Arnberg, Eriksson, Hultman, & Lundin, 2011; Arnberg, Hultman, Michel, & Lundin, 2013).

### Developmental perspective

There is a growing interest in developmental issues, and particularly child abuse studies in the Baltic countries. We could find at least two large cross-cultural studies with results from Latvia and Lithuania included in child abuse prevalence studies. Sebre et al. (2004) reported findings from a study of 1,145 adolescents aged 10–14 from Latvia, Lithuania, Macedonia, and Moldova. Parental violence was related with higher alcohol consumption and children from rural areas were experiencing more violence (Sebre et al., 2004). Another study from the eight Central and Eastern European countries (including Lithuania and Latvia) with 10,696 participants focused on the effects of childhood adversities in young adults aged 18–25 (Bellis et al., 2014). A study found that at least half of young adults experienced traumatic experiences in childhood, and at least four adversities in childhood significantly predicted mental health problems in adulthood (Bellis et al., 2014). A recent study published by Daugirdaitė, Van den Akker, and Purewal (2015) on PTSD and termination of pregnancy expands developmental perspective of traumatic stress research. A systematic review of 48 studies showed that PTSD after reproduction loss was predicted by previous traumatic experiences, other mental health problems, and advanced pregnancy (Daugirdaitė et al., 2015).

## Future directions of trauma research and practice in the Baltic States

Our brief review reveals the growing interest in traumatic stress in the Baltic countries. Psychotraumatology research is related to the specific socio-historical context of the region, with a strong interest on the political oppression effects. A majority of traumatic stress research from the Baltic States is fundamental, with a focus on mediating factors and resilience. While we found some interesting studies from the Baltic region, we should acknowledge that small numbers of traumatic stress studies has been published (e.g., in comparison to the Netherlands, Vermetten & Olff, 2013). There is a lack of epidemiological data, there are no published RCT PTSD treatments or early intervention studies, and PTSD neuroscience studies are not available from the Baltic States. However, we should also point out that our overview included only published studies and doctoral theses, and unpublished studies or studies in progress might be overlooked in this paper.

We could also identify a few important future directions for the traumatic stress research in the Baltic States. We found only limited data published on the epidemiology of trauma and PTSD in the region. Further studies are needed to acquire more data on the prevalence of stress-related disorders in the Baltic States. Longitudinal studies with a focus on trauma and resilience would be very welcomed. Trauma-focused treatments for PTSD and other stress-related disorders should be addressed more in research. The dissemination of trauma-focused evidence-based treatments is important for the development of psychotraumatology in the region. Early intervention, treatment, and healthcare utilization studies are important for the region because it could facilitate the availability of treatment for trauma-affected populations and also contribute to the development of psychosocial disaster interventions.

The field of psychotraumatology in the Baltic countries has emerged only after the collapse of the Soviet Union and the significant social changes that followed. Unfortunately, political oppression and wars are not unique for the Baltic States, but they share similar history with other Central and Eastern European post-communist countries. However, taking into account the specific social context and the historical path of trauma research, we can also identify that the developments and challenges of the trauma field in the Baltic countries are in line with psychotraumatology in other European countries (Şar, 2015). The complicated history of the region, successful adjustment to rapid social changes, and diversity of cultures could inspire new studies that could provide new insights about resilience and expand our understanding on the role of cultural and social factors of trauma.
